# Biodiversity and Conservation of Marine Mollusks in the Indo‐Pacific Convergence Zone

**DOI:** 10.1002/ece3.72364

**Published:** 2025-11-12

**Authors:** Xianshui Lai, Tingting Li, Bailin Cong, Pipit Pitriana, Bei Wang, Linlin Zhao, Shenghao Liu

**Affiliations:** ^1^ Marine Ecology Research Center First Institute of Oceanography, Ministry of Natural Resources Qingdao China; ^2^ School of Advanced Manufacturing Fuzhou University Fuzhou China; ^3^ Research Center for Biosystematics and Evolution, Research Organization for Life Sciences and Environment National Research and Innovation Agency Cibinong Indonesia

**Keywords:** anthropogenic pressure, conservation gap, marine protected areas, mollusk diversity, priority conservation areas, spatial distribution

## Abstract

The Indo‐Pacific Convergence Zone (IPCZ), a global marine biodiversity hotspot, faces increasing threats from anthropogenic activities and climate change. This study presents the first comprehensive assessment of mollusk diversity, identifies conservation gaps, and prioritizes protection areas in the IPCZ. Using 47,097 occurrence records of 3215 mollusk species, along with habitat and environmental data, we mapped biodiversity indices and integrated Marine Protected Areas (MPAs) and fishing effort. The results revealed significant spatial variability, with 11 priority conservation areas located in the Philippines, Indonesia, Papua New Guinea, and Australia. These areas contained 47.9% of mollusk species and 58.9% of threatened taxa, yet only 18.7% overlapped with existing MPAs. High fishing pressure disproportionately affected priority zones, particularly near Australia's Great Barrier Reef and Indonesia's Papua Province. Environmental factors, especially primary productivity and bathymetry, were key drivers of biodiversity, while rising temperatures had negative effects. Biogenic habitats, which cover 28% of the IPCZ, strongly correlated with mollusk diversity, with multi‐habitat zones supporting 42.3% of biodiversity. This study underscores the urgent need to expand protection in high‐diversity habitats and reduce fisheries pressure, providing actionable insights to enhance conservation strategies and ecosystem resilience in the IPCZ.

## Graphical abstract

An integrated assessment has identified 11 priority mollusk conservation areas in the Indo‐Pacific Convergence Zone, which harbor 47.9% of species and 58.9% of threatened taxa, though only 18.7% have MPA coverage. Multi‐habitat zones, such as coral–mangrove areas, support 42.3% of molluscan diversity. Fishing pressure overlaps 72.6% of unprotected priority areas, highlighting the need for urgent conservation action.

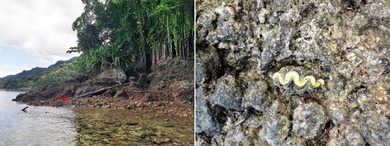

## 1 Introduction

The Indo‐Pacific Convergence Zone (IPCZ), spanning the tropical waters of the Indian and western Pacific Oceans, is one of the most biodiverse marine regions in the world, harboring 76% of the world's coral species, 58% of tropical mollusks, and 37% of reef fish species (Asaad et al. 2018; Brooks et al. 2006; Roberts et al. 2002). This region serves as both a “cradle” and “museum” of marine life, where geological history, ocean currents, and habitat heterogeneity have synergistically driven species origination, preservation, and overlap (Asaad et al. 2018; Li et al. 2021). Mollusks, the second‐largest animal phylum, dominate benthic communities and play pivotal roles in the ecosystems of the Indo‐Pacific Convergence Zone (IPCZ) (Bellwood and Meyer 2009; Paulay and Meyer 2006; Roberts et al. 2002). Particularly, over half of the approximately 450 new mollusk species described annually are discovered in the IPCZ, yet over 50% of coral‐associated mollusks face extinction risks from climate‐driven habitat degradation and overexploitation. Additionally, the IPCZ hosts a significant proportion of critical ecosystems including coral, mangrove, and seagrass (Briggs and Bowen 2013; Veron et al. 2009), which serve as important shelters and habitats for mollusks.

Mollusks play a vital role in maintaining ecosystem stability, facilitating nutrient cycling, and supporting species diversity (Gutiérrez et al. 2003; Oehlmann and Schulte‐Oehlmann 2003). Owing to their high sensitivity to environmental changes, they are commonly used as key indicator taxa for assessing marine ecosystem health and providing essential insights for dynamic ecosystem monitoring (Oehlmann and Schulte‐Oehlmann 2003). However, mollusks are increasingly threatened by habitat degradation and population declines resulting from intensified human activities and pronounced environmental fluctuations driven by climate change (Habibullah et al. 2022; McClanahan et al. 2008; Polidoro et al. 2010; Seto et al. 2023; Unsworth and Cullen 2010; Xu et al. 2024). These declines jeopardize not only ecosystem structure and function but also the stability of marine biodiversity at both regional and global scales. Therefore, urgent and effective conservation measures are essential to ensure the long‐term sustainability of molluscan diversity in the region.

Marine Protected Areas (MPAs) are widely recognized as effective tools for the conservation and management of marine biodiversity (Grorud‐Colvert et al. 2021; Venter et al. 2014). By restricting human activities in ecologically sensitive areas, MPAs play a crucial role in safeguarding both species and habitats (Asaad et al. 2018; Beger et al. 2003; Carminatto et al. 2025). In 2022, the 15th Conference of the Parties to the Convention on Biological Diversity adopted the Kunming‐Montreal Global Biodiversity Framework, which set the “30 × 30” target, aiming for at least 30% of the world's marine areas to be designated as protected by 2030. As of now, over 30 million km^2^ of the global ocean have been designated as MPAs, reflecting a more than tenfold increase over the past decade (UNEP‐WCMC 2025). However, despite the ongoing expansion of global MPAs coverage, only 8.3% of the ocean area is currently under protection, with just 2.9% classified as fully or highly protected (Pike et al. 2024). Achieving the global marine biodiversity conservation targets will necessitate the identification of priority areas and the establishment of larger, more effective MPAs (Butchart et al. 2015; Liu et al. 2023; Ricketts et al. 2005; Wang et al. 2023). The United Nations adopted the Agreement under the United Nations Convention on the Law of the Sea on the Conservation and Sustainable Use of Marine Biological Diversity of Areas beyond National Jurisdiction (BBNJ Agreement) on June 19, 2023 (United Nations 2025). This Agreement enables the establishment of legally protected MPAs in international waters that were previously unprotected (Miao and Chen 2025; Wang and Pan 2025). Its goal is to ensure the long‐term conservation and sustainable use of marine biodiversity in the high seas, contributing to global biodiversity objectives, supporting ecosystem resilience, and promoting equitable participation in ocean governance (Huffman 2025; Morgera 2025; Wang 2025). Traditionally, the identification of priority conservation areas has primarily relied on species richness (Caldecott et al. 1996; Pompa et al. 2011; Trebilco et al. 2011). However, conservation planning based solely on species richness may be inadequate for providing a systematic framework for biodiversity protection. Furthermore, global conservation strategies often overlook critical habitats (Caldecott et al. 1996; Pompa et al. 2011; Trebilco et al. 2011), and the increasing intensity of fishing pressure has, to some extent, reduced the effectiveness of certain MPAs (Zhang et al. 2024).

Although mollusks represent one of the most widely distributed groups within the Indo‐Pacific Convergence Zone (Hoeksema 2007), research on their conservation remains limited, hindering the development of science‐based management strategies. A critical initial step in addressing this gap is to establish a comprehensive understanding of their biodiversity patterns. Toward this goal, we employed three key metrics: sampling effort, species richness, and the Shannon‐Wiener index. Specifically, sampling effort reflects the intensity of biological surveys conducted across regions and serves as a baseline for assessing potential biases and uneven coverage, thereby improving the reliability of biodiversity interpretations (Roy‐Dufresne et al. 2019). Species richness denotes the number of distinct species or taxonomic groups within a given community or area and enhances ecosystem functioning (Barrientos‐Luján et al. 2021). The Shannon‐Wiener index, which integrates both richness and evenness, offers further insights into community structure and the degree of species dominance (Luo et al. 2025). Collectively, these metrics provide a balanced framework for identifying biodiversity hotspots and conservation gaps that may remain undetected when relying on a single measure. Using 47,097 occurrence records of 3215 mollusk species and data on key habitats such as coral reefs, mangroves, and seagrass, this study mapped the spatial distribution of sampling effort, species richness, and the Shannon‐Wiener index. We identified biodiversity hotspots and delineated priority conservation areas. By integrating data on existing MPAs and fishing effort, we assessed the effectiveness of current conservation measures and identified gaps in mollusk protection. Our findings provide a scientific foundation for the long‐term conservation and sustainable management of molluscan diversity in the IPCZ and support broader global marine ecosystem sustainability efforts.

## 2 Materials and Methods

### 2.1 Study Area

**FIGURE 1:**
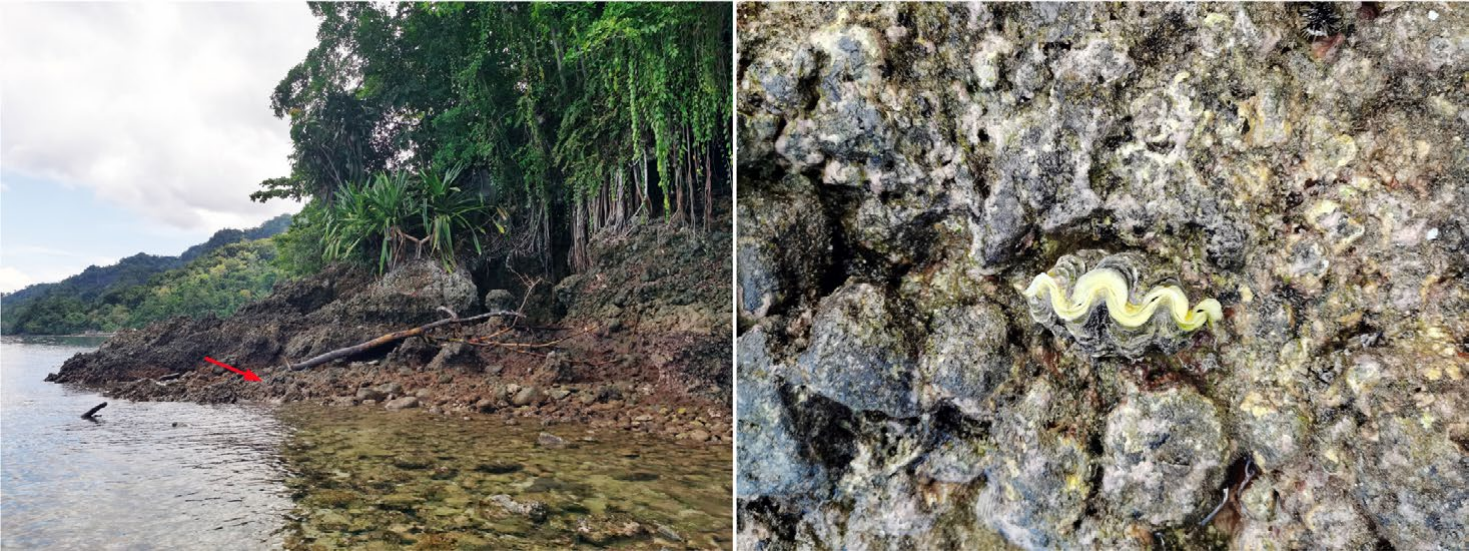
Intertidal habitat between mangrove and coral reef ecosystems, with a giant clam indicated by the red arrow (location: Maluku Islands, Indonesia; November 21, 2024).

From November 15 to 27, 2024, a joint research expedition was conducted by the First Institute of Oceanography, Ministry of Natural Resources of China, and the Research Organization for Life Sciences and Environment under Indonesia's National Research and Innovation Agency (BRIN), in the waters of Biru Bay, Ambon City, Indonesia. The mission focused on assessing marine biodiversity, with particular emphasis on coral reefs and intertidal habitats, from which mollusk samples were collected alongside other taxonomic groups (Figure 1). Preliminary observations from the expedition reveal that the region supports exceptionally high levels of biodiversity. However, it also faces growing pressures from human activities, including fisheries operations and climate change. Notably, giant clams (Tridacnidae) are legally protected in China due to their conservation status. Motivated by these findings, we seek to investigate molluskan diversity and identify conservation gaps across a broader geographical context.

**FIGURE 2:**
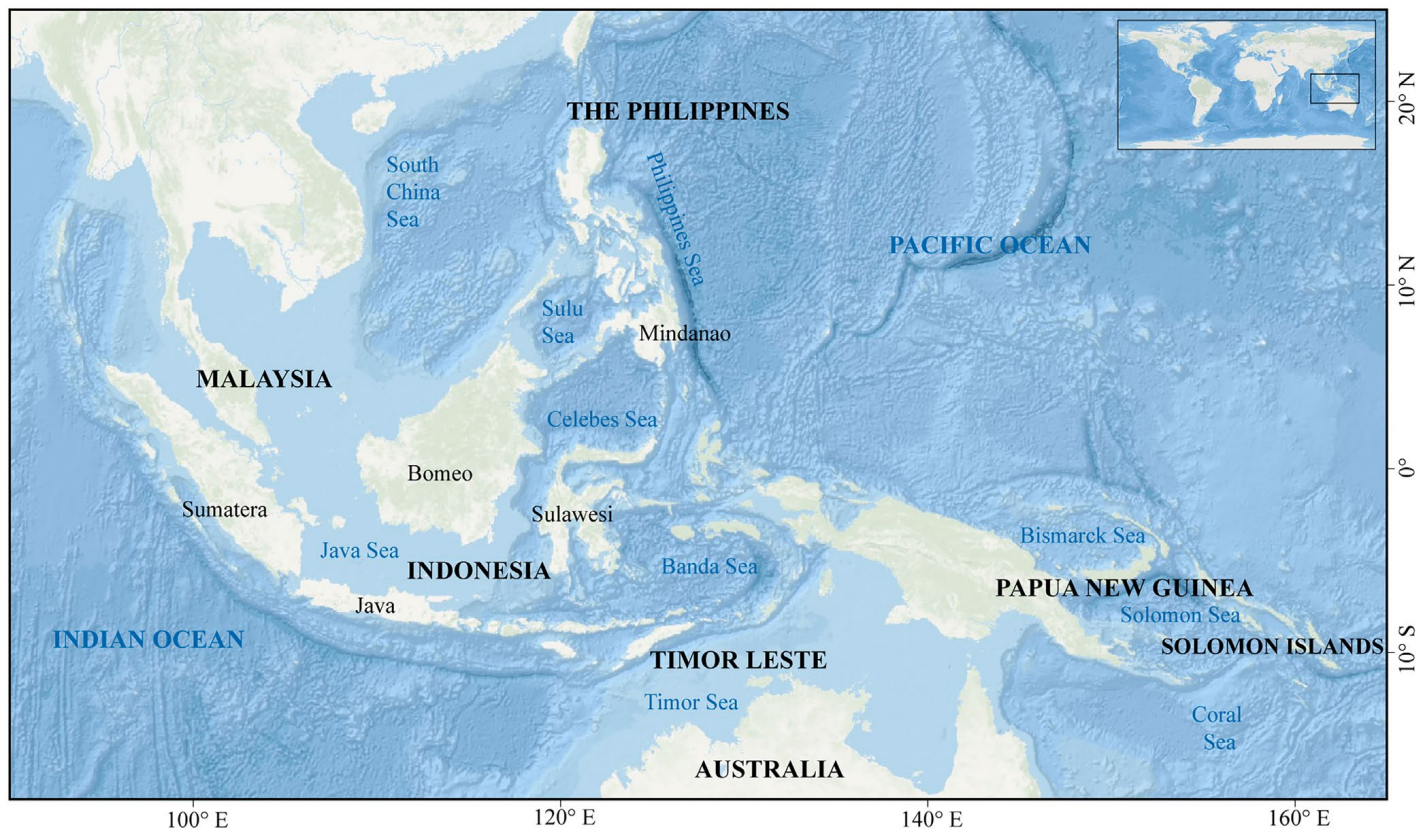
Schematic map showing the boundaries of the Indo‐Pacific Convergence Zone (18° S–25° N, 90° E–165° E).

In this study, the research domain encompassed the entire Indo‐Pacific Convergence Zone (IPCZ), extending from 18° S to 25° N latitude and 90° E to 165° E longitude (Figure 2). The region is characterized by an extensive shallow continental shelf, marginal seas, and a high density of islands. It fully incorporates exclusive economic zones of Indonesia, Malaysia, Papua New Guinea, the Philippines, Solomon Islands, and Timor Leste, while also incorporating adjacent maritime areas of other nations, including Australia.

### 2.2 Data Collection and Processing

Species occurrence records for mollusks were obtained from the Global Biodiversity Information Facility (GBIF, https://www.gbif.org/) and the Ocean Biogeographic Information System (OBIS, https://www.iobis.org/). To ensure temporal relevance, we restricted the dataset to records collected between 2000 and 2024. The geographic distribution data were subsequently merged and subjected to rigorous quality control procedures. Data cleaning was performed using the R package robis for correcting coordinate errors and removing duplicate entries to improve both accuracy and reliability (Provoost 2018). Additionally, terrestrial and fossil records were excluded to maintain consistency with the current research objectives. Species names were cross‐referenced and validated against the World Register of Marine Species (WoRMS) to ensure taxonomic precision (WORMS).

To identify threatened species, we consulted the IUCN Red List of Threatened Species (https://www.iucnredlist.org), which classifies species into categories including Critically Endangered (CR), Endangered (EN), Vulnerable (VU), Data Deficient (DD), Near Threatened (NT), and Least Concern (LC). Additionally, we referred to Appendices I and II of the Convention on International Trade in Endangered Species of Wild Fauna and Flora (CITES) to compile a list of threatened and potentially threatened mollusk species. Appendix I includes species threatened with extinction and potentially affected by international trade, while Appendix II lists species that are not currently threatened but may become so if trade is not properly regulated. A comprehensive list of the threatened and potentially threatened species identified in this study is provided in Table S1.

Spatial distribution data for three key marine habitats—coral, seagrass, and mangroves—were also included in the analysis. These datasets were sourced from the UNEP World Conservation Monitoring Centre (UNEP‐WCMC, https://data.unep‐wcmc.org/). Upon integrating the data, the centroid algorithm in the QGIS platform was applied to convert polygon habitat layers into point‐based latitude and longitude coordinates for spatial analysis. All spatial data were projected using the WGS84 geographic coordinate system to ensure consistency and accuracy in geospatial analyses. Following these procedures, a total of 47,097 occurrence records for marine mollusks were obtained, representing 3215 species; 7374 occurrence records for threatened mollusk species, encompassing 235 species; and 21,705 point records for biogenic habitats. All datasets were deduplicated to enhance accuracy and reliability.

To analyze the influence of environmental factors on biodiversity distribution patterns, we extracted 11 key variables from Bio‐ORACLE v3.0 (https://www.bio‐oracle.org). These variables included ocean temperature (°C), bathymetry (m), seawater velocity (m·s^−1^), salinity (psu), silicate (mol·m^−3^), phosphate (mmol·m^−3^), primary productivity (mmol·m^−3^), pH, dissolved molecular oxygen (mmol·m^−3^), nitrate (mmol·m^−3^), and iron concentration (mmol·m^−3^). For each mollusk occurrence record, we obtained the corresponding environmental values and then calculated the average of each variable within 1‐degree latitude and longitude grid cells using the QGIS platform. This approach ensured spatial consistency and comparability of the dataset.

To assess conservation gaps for mollusks in the Indo‐Pacific Convergence Zone (IPCZ), this study integrated existing marine protected areas (MPAs) and fisheries catch hotspots to conduct a conservation gap analysis of priority mollusk conservation areas. Data on established MPAs were obtained from the World Database on Protected Areas (WDPA, https://www.protectedplanet.net/). Fisheries effort data for the IPCZ from 2021 to 2023 were sourced from Global Fishing Watch (http://globalfishingwatch.org/). Fishing effort was quantified using fishing hours as an indicator to evaluate the potential threat posed by fisheries activities to marine biodiversity.

### 2.3 Spatial Distribution Characteristics of Mollusks and Their Habitats

The mollusk distribution data within the study area were statistically analyzed at the class level. Distribution maps for each mollusk class were generated to systematically examine the classification structure and spatial distribution characteristics of mollusks. A 1‐degree latitude and longitude grid was established across the Indo‐Pacific Convergence Zone (IPCZ) using R, with each full grid cell covering approximately 12,321 km^2^, as 1° corresponds to approximately 111 km. A total of 3225 grid cells were created to enable fine‐scale calculations of sampling effort (number of occurrence records), species richness (total number of species), and the Shannon‐Wiener index for mollusks, threatened mollusks, and biogenic habitats. These metrics were used to map their respective spatial patterns and were analyzed at the species level. Subsequently, Pearson correlation analysis was conducted among the three biodiversity indices across the three biological groups to assess their interrelationships.

### 2.4 Priority Conservation Areas Identification

To avoid analytical bias from duplicate data, all records of threatened species were first excluded from the mollusk dataset to construct a non‐threatened species dataset. Using a 1° × 1° latitude and longitude grid, we quantified sampling effort, species richness, and the Shannon‐Wiener index for non‐threatened mollusks, threatened mollusks, and biogenic habitats. These metrics were then weighted and integrated to identify corresponding biodiversity hotspots.

The specific procedures were as follows. First, the sampling effort metrics of the three biological groups were normalized. Based on their ecological relevance, a weighted summation was then conducted to generate a composite sampling effort index. According to expert consultation, the assigned weights were 35% for the non‐threatened mollusk dataset, which reflects the overall distribution of the mollusk community but includes many species of lower conservation priority; 45% for the threatened mollusk dataset, which is relatively sparse but holds greater conservation value due to the higher extinction risk; and 20% for the biogenic habitat dataset, which represents environmental features favorable to mollusk survival, though its broad ecological nature warranted a lower weight. Based on the resulting composite index, Hot Spot Analysis (Getis‐Ord Gi*) was conducted using the spdep and sf packages in R to identify statistically significant sampling effort hotspots at the 99%, 95%, and 90% confidence levels. Z‐scores were used to evaluate statistical significance (Asaad et al. 2018). The same method was applied to species richness and Shannon‐Wiener index layers to determine their respective hotspot distributions.

The Getis‐Ord GI analysis was performed using the R packages spdep and sf to identify spatial clusters of biodiversity importance at the regional scale. The analysis evaluates the spatial association of high or low values by comparing the local sum of a focal grid cell and its neighbors to the global sum of all grid cells, generating a statistically significant Z‐score (GI statistic). A significantly positive Z‐score indicates a cluster of high values (hotspots), with larger Z‐scores representing stronger clustering intensity. Conversely, a significantly negative Z‐score indicates a cluster of low values (cold spots), with smaller Z‐scores corresponding to greater aggregation of low values. The hotspot analysis identified three levels of hotspots (at 99%, 95%, and 90% confidence), three levels of cold spots at the same confidence levels, and one category of non‐significant clusters (Asaad et al. 2018). In this study, only hotspots were considered. To examine the relationships among the three composite biodiversity indices, linear regression models were applied. Finally, priority conservation areas for mollusks in the Indo‐Pacific Convergence Zone were identified by integrating the hotspots of sampling effort, species richness, and the Shannon‐Wiener index at the 99% confidence level (Malczewski 2006).

### 2.5 Analysis of Environmental Factor Impacts

To evaluate the influence of environmental variables on the composite biodiversity index, this study applied the Extreme Gradient Boosting (XGBoost) model for prediction and used SHapley Additive exPlanations (SHAP) values to quantify the contribution of each environmental factor. XGBoost is an efficient machine learning algorithm based on gradient boosting that can effectively capture complex nonlinear relationships between environmental factors and biodiversity. In this analysis, biodiversity was treated as the response variable, and environmental variables were used as predictors. The model was trained using Gradient Boosting Decision Trees with hyperparameter tuning to enhance its generalization performance (Ghafarian et al. 2022). To further explore the effects of environmental factors on biodiversity, SHAP value analysis was employed. This method is grounded in Shapley value theory and assigns a contribution score to each feature for individual predictions. It reveals both the relative importance of each environmental factor in predicting biodiversity and the direction of its influence. SHAP analysis not only quantifies the relative contributions of environmental factors but also provides interpretable visualizations of their effects, thereby offering a robust scientific basis for understanding the relationship between environmental variables and biodiversity.

### 2.6 Conservation Gap Analysis

To evaluate the conservation effectiveness for mollusks, this study conducted a conservation gap analysis by integrating existing Marine Protected Areas with fisheries effort hotspots, focusing on the identified priority conservation areas for mollusks. To understand the extent of fishing pressure on mollusk species, fisheries data were first cleaned and aggregated to obtain monthly fishing effort measured in operation hours. The specific procedures included the following. First, monthly fishing operation hours were aggregated using the time range field, while different types of fishing activities were classified based on the gear type field. Based on this classification, distribution maps were generated for mollusk‐targeted fishing pressure, non‐mollusk fishing pressure, and ambiguous fishing effort. Second, the total annual fishing effort targeting mollusks in 2023 was calculated and summarized within grid cells measuring 1 degree in both latitude and longitude. Raster layers were then generated by applying a logarithmic transformation to the effort data. Third, fishing effort hotspots were defined as the top 20% of areas in terms of fishing intensity, representing potential high‐risk zones where fishing activities may threaten marine biodiversity. After identifying fisheries effort hotspots, the Marine Protected Area layer was overlaid with the priority conservation areas for mollusks. Based on this integration, the following analyses were conducted. First, the extent to which mollusk priority conservation areas are currently covered by existing Marine Protected Areas was assessed to identify ecologically important regions that are insufficiently protected in the current protection network. Second, fishing activities within mollusk priority areas were analyzed to examine their spatial relationship with Marine Protected Areas.

## 3 Results

### 3.1 Biodiversity Distribution

**FIGURE 3:**
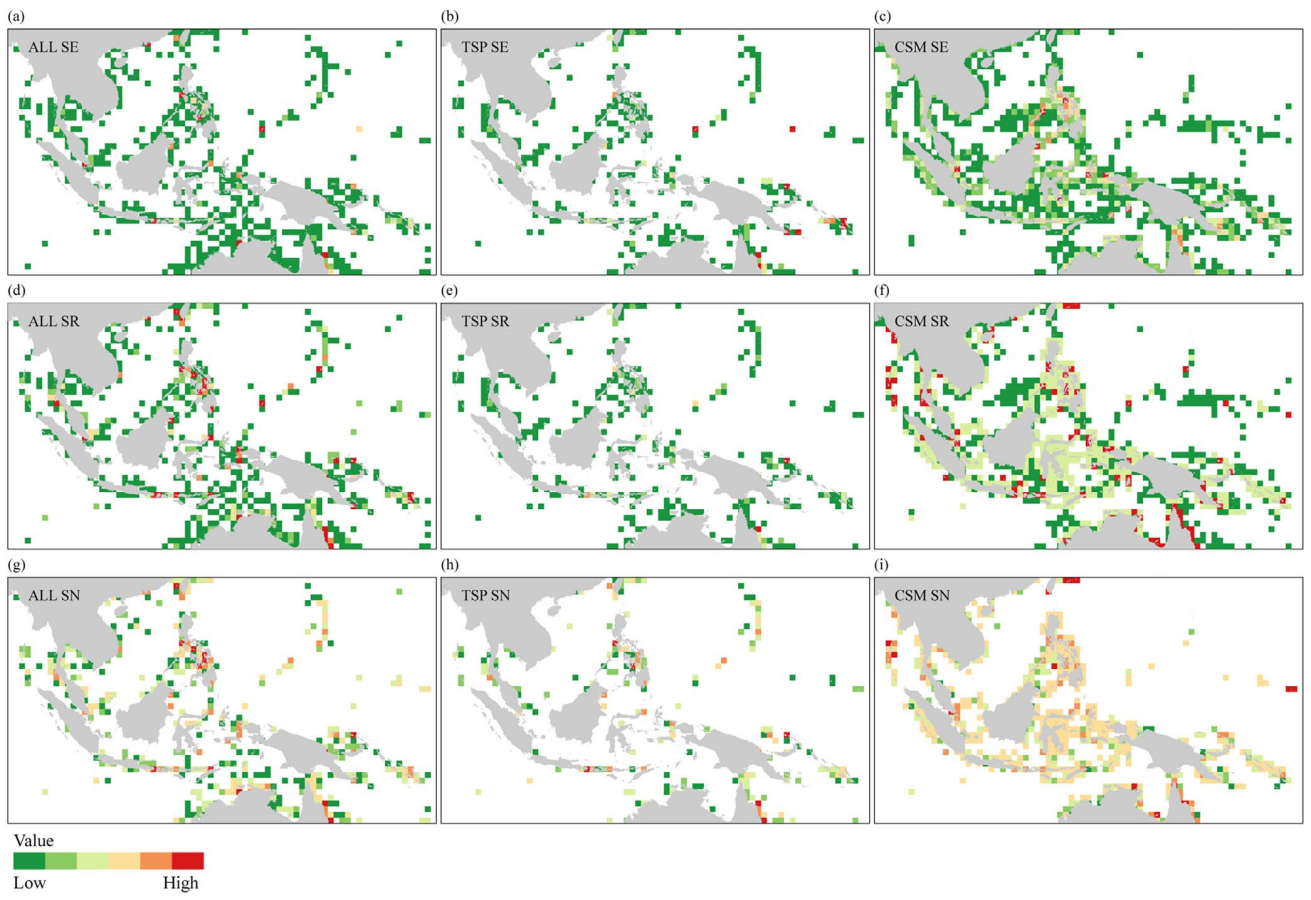
Distribution patterns of mollusks and biogenic habitats in Indo‐Pacific Convergent Zone according to sampling effort (SE), species richness (SR), and Shannon‐Wiener index (SN). (a) SE of all molluscan species (ALL SE), (b) SE of threatened molluscan species (TSP SE), (c) SE of coral, seagrass and mangrove (CSM SE), (d) SR of all molluscan species (ALL SR), (e) SR of threatened molluscan species (TSP SR), (f) SR of coral, seagrass and mangrove (CSM SR), (g) SN of all molluscan species (ALL SN), (h) SN of threatened molluscan species (TSP SN), and (i) SN of coral, seagrass and mangrove (CSM SN). The color gradient from green to red represents values ranging from low to high. Detailed values rang corresponding to different colors are available in the Figure S2.

A total of 47,097 mollusk occurrence records were obtained, with the majority belonging to the class Gastropoda, which covered 19 orders, followed by Bivalvia, Cephalopoda, Polyplacophora, and Scaphopoda (Figure S1). Mollusk distributions covered about 16.12% of the total region within the study area (Figure 3a), which was calculated based on the number of grid cells containing mollusk occurrences relative to the total number of grid cells. Areas with high sampling effort for mollusks were mainly located around Mindanao in the Philippines, Java in Indonesia, southern Malaysia, the surrounding waters of Palau, the outer eastern edge of the Great Barrier Reef, and the southeastern Timor Sea. Luzon Island in the Philippines exhibited the highest sampling effort, with 4602 records (Figure 3a–c). Regions with high species richness were broadly distributed across the marine areas of Indonesia, the Philippines, Papua New Guinea, Australia, Malaysia, the South China Sea near China, and the Solomon Islands. In particular, the southern region of Luzon Island displayed exceptionally high species richness, with a single grid cell recording 741 species (Figure 3d–f). Areas with high Shannon‐Wiener index values were concentrated in central and northern Philippines, eastern Papua New Guinea, southwestern Timor‐Leste, the western Indonesian archipelago, and northeastern Australia. Among these, Luzon Island recorded the highest Shannon‐Wiener index value of 5.84 (Figure 3g–i).

A total of 7375 distribution points associated with threatened mollusks were identified, accounting for 15.7% of the overall mollusk distribution points and representing 236 species. The largest group was Gastropoda, with 148 species, followed by Cephalopoda (73 species), Bivalvia (14 species), and Polyplacophora (1 species). Based on the criteria of the International Union for Conservation of Nature (IUCN) Red List, the threatened mollusk species identified in this study included 4 species classified as Critically Endangered, 8 as Endangered, 1 as Vulnerable, 4 as Near Threatened, 173 as Least Concern, and 36 as Data Deficient (Table 1). Furthermore, 12 species were listed under CITES Appendix II.

**TABLE 1:** Number of threatened species based on the IUCN Red List and CITES.

Category	Gastropoda	Bivalvia	Cephalopoda	Polyplacophora	Scaphopoda
**IUCN Red List**					
Critically endangered	3	1	—	—	—
Vulnerable	1	—	—	—	—
Endangered	7	1	—	—	—
Near threatened	2	—	1	1	—
Least concern	126	2	45	—	—
Data deficient	9	2	25	—	—
**CITES**					
Appendix II	—	8	4	—	—

*Note:* Within the class Cephalopoda, one species is simultaneously listed as Data Deficient and included in CITES Appendix II, while another species is simultaneously listed as Near Threatened and included in CITES Appendix II.

Regions with high sampling efforts for threatened mollusk species were primarily concentrated in eastern Papua New Guinea, eastern Australia, and the Solomon Islands (Figure 3b). Areas exhibiting relatively high species richness included Java (Indonesia), northeastern Papua New Guinea, and regions near the Great Barrier Reef (Australia), with species richness ranging from 42 to 62 species (Figure 3e). Elevated Shannon‐Wiener index values were observed in Luzon (Philippines), northeastern Australia, the Bismarck Sea, and southwestern Java (Figure 3h). For protected species, the maximum number of occurrence records within a single grid cell was 1484, with peak species richness of 62 species and a maximum Shannon‐Wiener index of 3.56. A total of 88 grid cells contained only a single occurrence record, and 106 grid cells contained only one threatened species (Table S2). Approximately 9.5% of the IPCZ contained at least one protected mollusk species. Within the IPCZ, coral habitats were the most widespread, covering 23% of the region, followed by mangroves (19.7%) and seagrass (3.8%). Biogenic habitats accounted for approximately 28% of the IPCZ area. Of these, 44.1% of grid cells were dominated by a single habitat type, 45.5% by two combined habitat types, and only 10.4% by all three. Coral–mangrove combinations accounted for 42.3% of the total biogenic habitat coverage (Table S2).

Regions with high biogenic habitat sampling effort were primarily located in the Sulu Sea, Mindanao, and Luzon Islands in the Philippines, southern Gulf of Papua, eastern Papua New Guinea waters, southwestern West Papua, and Sulawesi Island, Indonesia (Figure 3c). Although areas where coral, seagrass, and mangrove biogenic habitats co‐occurred were scattered, they were found along the coastlines of nearly all the countries within the study region (Figure 3f). For regions with high biogenic habitat Shannon‐Wiener index values, these were mainly located on the eastern Taiwan coast, southern Malaysia, northern Luzon and Mindanao Islands in the Philippines, Sulu Sea, northern Timor Island, western and southern West Papua, Sulawesi Island, and northeastern Australia (Figure 3i).

**FIGURE 4:**
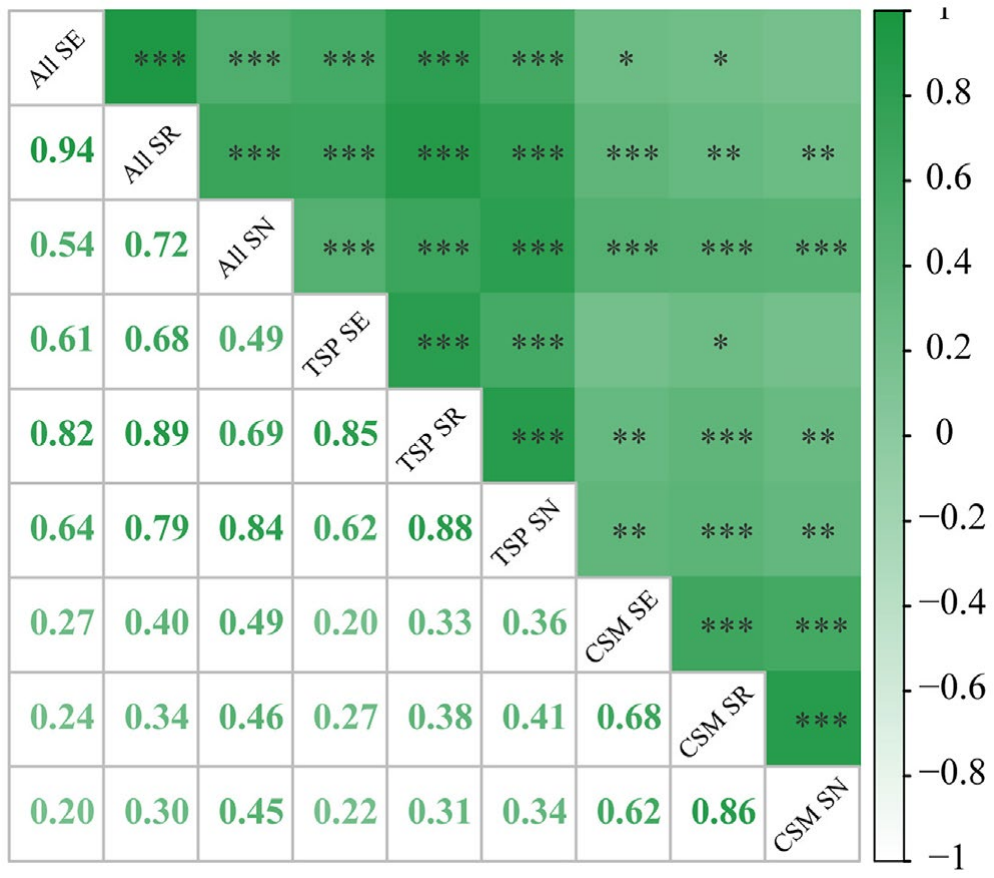
Pearson correlation between sampling effort (SE), species richness (SR), and Shannon‐Wiener index (SN) for mollusks (ALL), threatened mollusks (TSP), and biogenic habitats (CSM).

Overall, most biodiversity indices exhibited significant positive correlations, particularly between sampling effort and species richness, as well as between species richness and community evenness (Figure 4). Although the correlations between biogenic habitat diversity indices and both overall and threatened mollusks were generally weaker, most were statistically significant. Specifically, CSM SE was positively correlated with All SR (*r* = 0.40) and All SN (r = 0.49), while CSM SR showed moderate correlations with All SN (*r* = 0.46) and TSP SR (r = 0.38). In addition, CSM SN was positively correlated with All SN (*r* = 0.45), and CSM SR with TSP SN (r = 0.41); both correlations reached a highly significant level (*p* < 0.001, ***). These results suggest that the diversity of key biogenic habitats might have played a positive role in maintaining overall species diversity and might have been particularly important in supporting the biodiversity of threatened mollusks.

### 3.2 Priority Conservation Areas

**FIGURE 5:**
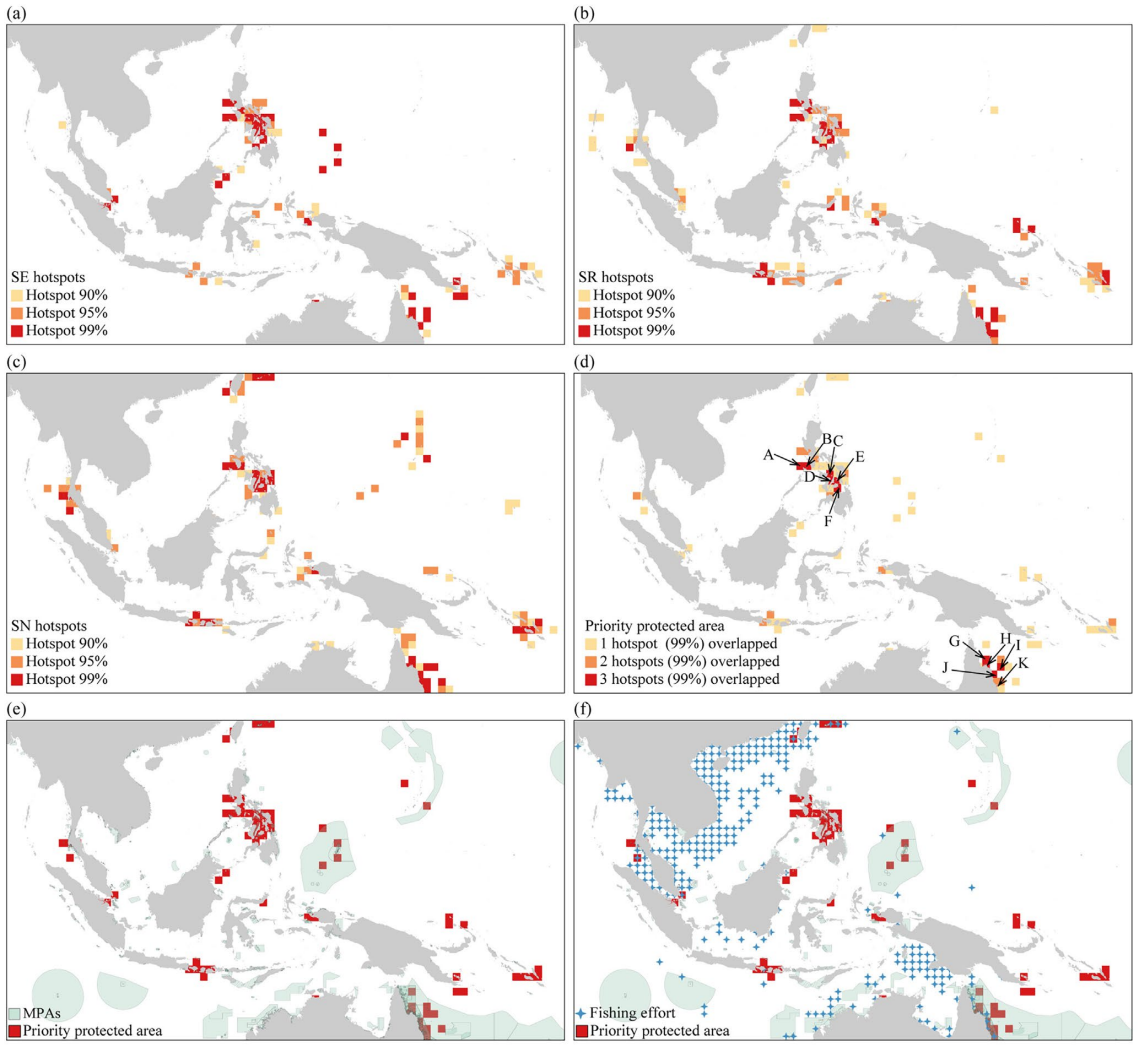
Distribution of biodiversity hotspots and priority protected areas. (a) sampling effort (SE) hotspots, (b) species richness (SR) hotspots, (c) Shannon‐Wiener index (SN) hotspots, (d) priority protected area identified by overlaying the hotspots (99%) of SE, SR, and SN, (e) overlap of marine protected areas (MPAs) with priority protected area, and (f) overlap of marine protected areas (MPAs) and 2023 top 20% of fishing effort with priority protected area. Key sites in (d) include: (A) northwestern Palawan Island, (B) western Mindoro Island, (C) western Negros Island, (D) western Cebu Island, (E) eastern Leyte Island, (F) Mindanao Island, (G) east of Kira, (H) southeast of Kira, (I) southeastern Santa Cruz Islands, (J) east of Makira Island, and (K) southeast of Makira Island.

**FIGURE 6:**
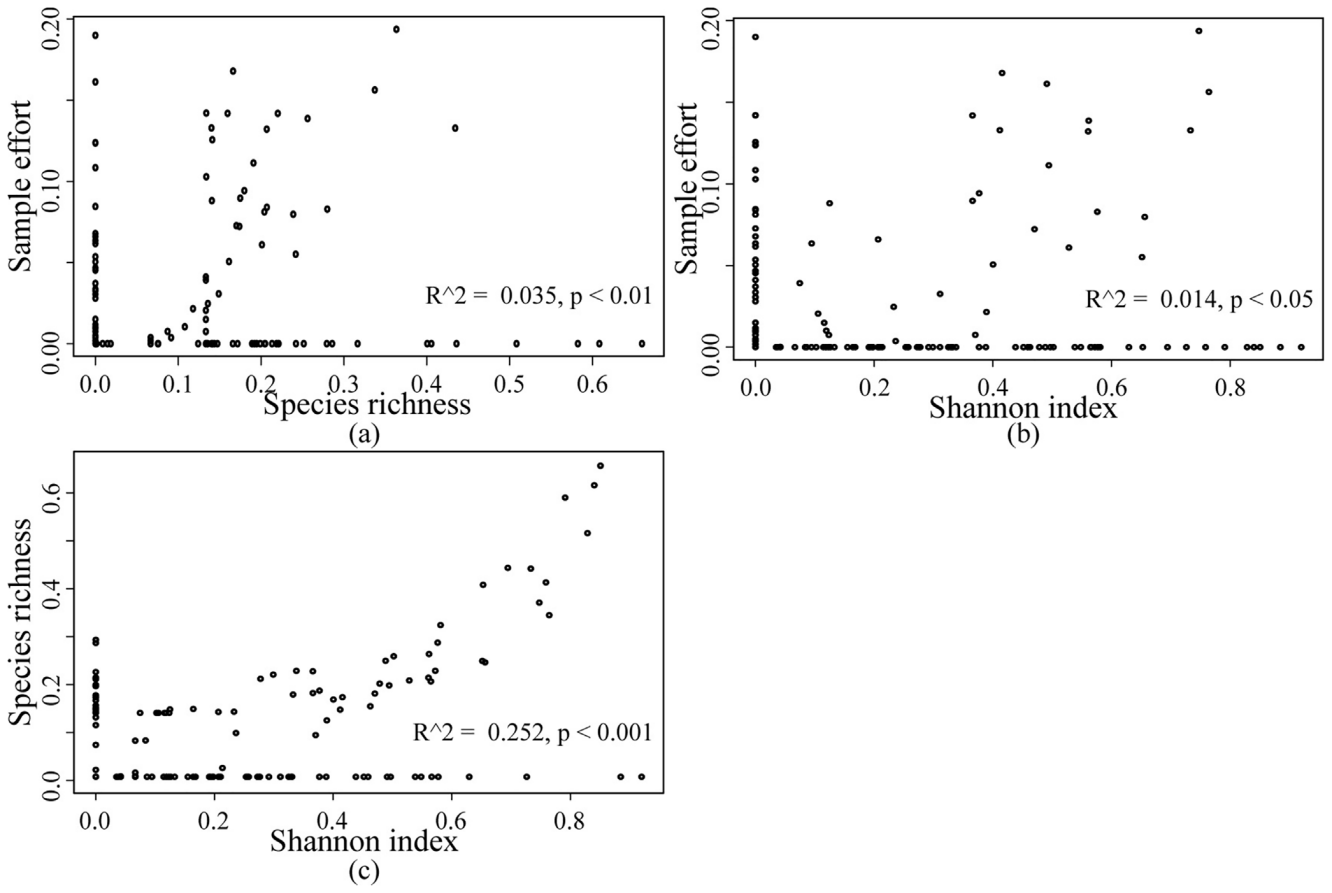
Relationship diagram of the biodiversity index of hotspot areas. (a) Distribution abundance vs. species richness; (b) Distribution abundance vs. Shannon‐Wiener index; (c) Species richness vs. Shannon‐Wiener index.

Approximately 2.33% of the IPCZ was clustered as sampling effort hotspots, with 0.53% at the 90% confidence level (*Z*_max_ = 1.95), 0.68% at the 95% confidence level (*Z*_max_ = 2.53), and 1.12% at the 99% confidence level (*Z*_max_ = 6.76) (Figure 5a). Approximately 3.13% of the IPCZ was classified as species richness hotspots, with 1.09% at the 90% confidence level (*Z*_max_ = 1.96), 0.99% at the 95% confidence level (*Z*_max_ = 2.57), and 1.05% at the 99% confidence level (*Z*_max_ = 7.94) (Figure 5b). Approximately 3.44% of the IPCZ was clustered as Shannon‐Wiener index hotspots, with 1.02% at the 90% confidence level (*Z*_max_ = 1.95), 1.18% at the 95% confidence level (*Z*_max_ = 2.57), and 1.24% at the 99% confidence level (*Z*_max_ = 6.62) (Figure 5c). Biodiversity hotspots with a 99% confidence level were classified as high hotspot clusters, referred to as core biodiversity hotspots. The analysis showed that the spatial distribution patterns of sampling effort, species richness, and Shannon‐Wiener index hotspots were weakly correlated (Figure 6), indicating limited mutual influence among different biodiversity indices. Therefore, to further identify key conservation areas, the core hotspot areas of the three biodiversity indices were overlaid, with the union of the 99% confidence level hotspots for all three indices defined as priority conservation areas (Figure 5d).

In the priority conservation areas, sampling effort hotspots were mainly concentrated in the Visayan Islands and Mindanao Island in the Philippines, the Philippine Sea, southern Singapore, the western Sulawesi Sea, the Northern Territory of Australia, and southeastern Papua New Guinea. Species richness hotspots were primarily distributed in the Visayan Islands, Luzon Island, the Andaman Sea, northeastern Sulawesi Island, Java Island, the Bismarck Sea, and the Solomon Islands. Meanwhile, the core distribution areas of Shannon‐Wiener index hotspots included the western and eastern Taiwan coasts, the Visayan Islands, the western Pacific, the eastern Andaman Sea, Papua Province in Indonesia, northeastern Australia, and the Solomon Islands. Further analysis of the spatial overlap of hotspots revealed that the overlapping areas between sampling effort hotspots and species richness hotspots included Luzon Island, Mindanao Island, West Papua Province in Indonesia, and northeastern Australia. The overlapping areas between species richness hotspots and Shannon‐Wiener index hotspots were mainly located in the Andaman Sea, the Visayan Islands, Java Island in Indonesia, Queensland and the Great Barrier Reef in Australia, and the Solomon Islands. By combining the overlapping areas of the three biodiversity hotspots, we ultimately identified 11 key priority conservation sites, six of which were in the Philippines: northwestern Palawan Island, western Mindoro Island, western Negros Island, western Cebu Island, eastern Leyte Island, and Mindanao Island. Additionally, five key sites were identified near the Solomon Islands, including east and southeast of Kira, east, southeast of Makira Island, and the southeastern part of the Santa Cruz Islands.

### 3.3 Environmental Factor Impacts

**FIGURE 7:**
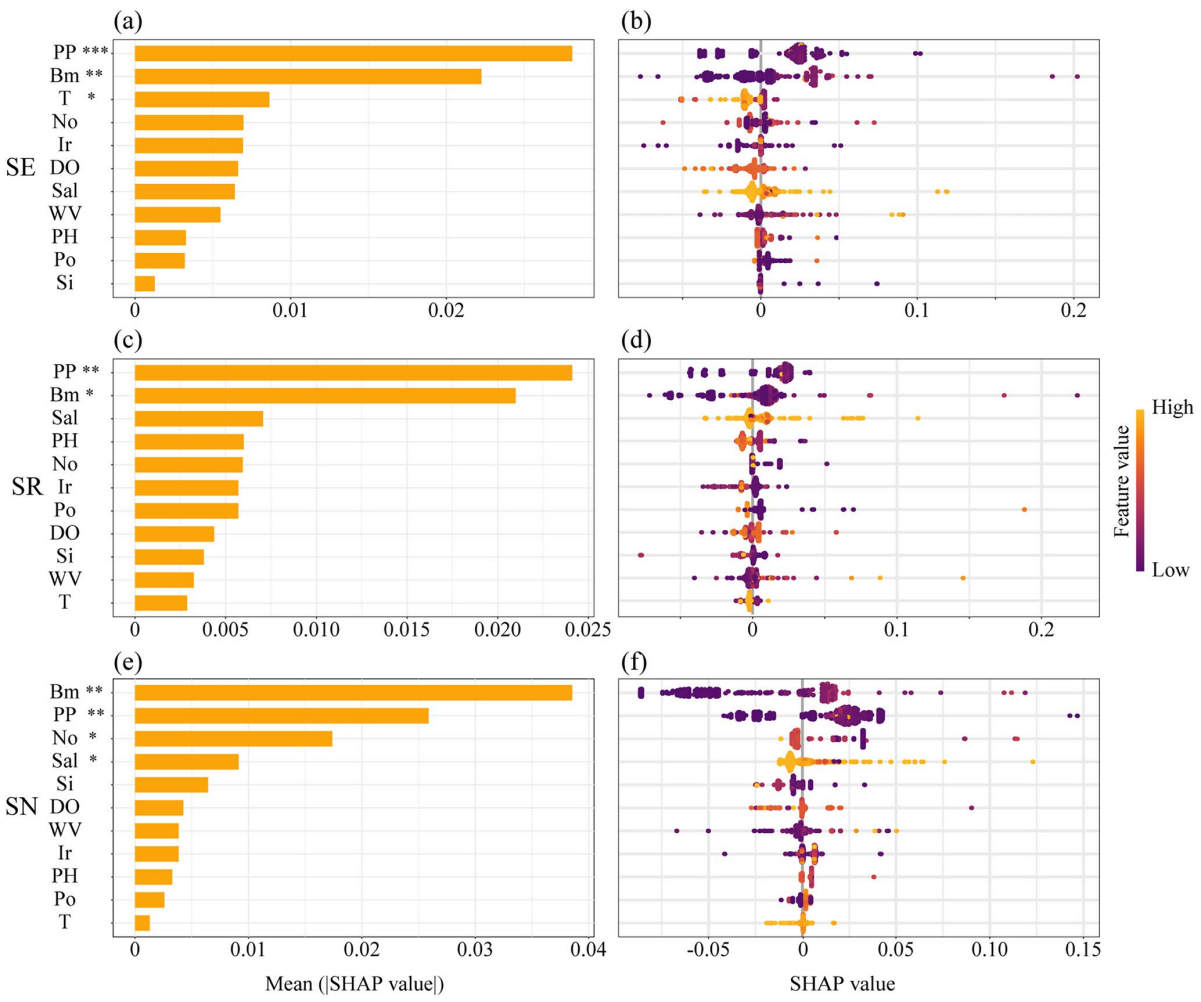
Shap mean values (left) and shap value distribution (right) of environmental factors on biodiversity indices in hotspot areas. Biodiversity indices in hotspot areas include (a, b) Sample effort, (c, d) species richness, and (e, f) Shannon‐Wiener index. Feature values are color‐coded, with yellow representing high values and purple representing low values. Significance levels of predictor contributions are indicated next to variable names (***p < 0.001, **p < 0.01, *p < 0.05). Bm, Bathymetry (m); DO, dissolved molecular oxygen (mmol/m^3^); Ir, iron (mmol/m^3^); No, nitrate (mmol/m^3^); Po, phosphate (mmol/m^3^); PP, primary productivity (mmol/m^3^); Sal, salinity (psu); Si, silicate (mmol/m^3^); T, ocean temperature (°C); WV, sea water velocity (m/s).

In the analysis of the effects of environmental factors on the different biodiversity hotspots, mean (|SHAP value|) and SHAP values were used to comprehensively assess the importance and contribution of environmental factors to the model predictions. Figure 7a,c,e displayed the mean (|SHAP value|), which measured the global importance ranking of each environmental factor. Figure 7b,d,f showed the SHAP values, reflecting the influence of each environmental factor on the species distribution. The x‐axis represented the size and direction (positive or negative) of each environmental factor's contribution to the prediction results. The analysis results indicated that primary productivity (PP) and bathymetry (Bm) were the most important environmental factors in all three biodiversity hotspot distribution patterns, consistently making core contributions to model predictions. In the analysis of the sampling effort hotspots (SE), the mean (|SHAP value|) of primary productivity (PP) reached 0.028, the highest among all factors, followed by bathymetry (Bm) at 0.022. In the species richness hotspots (SR), the values of PP and Bm were 0.024 and 0.021, respectively, further highlighting their key roles. In the Shannon‐Wiener index hotspots (SN), Bm had the highest value at 0.039, with PP closely following at 0.026. Additionally, temperature (T) had varying effects on different biodiversity. In the sampling effort hotspot analysis, temperature ranked third in contribution, while in the species richness and Shannon‐Wiener index hotspot analyses, its contribution was the smallest. However, it is noteworthy that temperature showed a negative effect in all three biodiversity hotspot distribution patterns (Figure 7b,d,f), indicating that an increase in temperature may have an adverse impact on the biodiversity distribution of mollusks.

### 3.4 Conservation Effectiveness and Gaps

In the analysis of fishing pressure in the study area, this research used the Global Fishing Watch global fishing effort data from 2021 to 2023 to calculate and map the status of marine fisheries (Figure S3). The results showed that after excluding unclear fishing pressure data, fishing activities on marine mollusks during 2021–2023 exhibited two notable characteristics: (1) High fishing pressure, with the proportion of mollusk catch steadily increasing. The proportion of fishing effort directed toward mollusks rose annually, exceeding 60% each year, with a peak of over 72% (61.28% in 2021, 65.60% in 2022, and 72.58% in 2023), indicating high intensity and a continuous growth trend in the exploitation of this resource. (2) Periodic fluctuations in fishing pressure, with the closed fishing season having a significant impact. During the closed fishing season (usually from spring to summer, calculated as May to July in this study), the fishing pressure on mollusks significantly decreased, and their share of the total fishing effort dropped to 54.06%, below the annual average level (66.49%). The fishing pressure on mollusks during the closed fishing season accounted for only 20.33% of the total mollusk fishing effort for the year, significantly lower than the 34.27% for non‐mollusk catches during the same period (May to July). Notably, in April 2021, the fishing pressure on mollusks reached as high as 74.34%, but dropped to 42.75% during the closed season. This trend suggested that the fishing ban policy had somewhat alleviated the fishing pressure on mollusks, but overall fishing intensity remained high.

In the protection gap analysis, existing Marine Protected Areas (MPAs) had a relatively wide coverage in the study area, but their distribution is uneven, with some regions having larger protected areas, while others had relatively small ones (Figure 5e). However, the coverage of MPAs over priority protection areas was quite limited, primarily concentrated in areas near the Great Barrier Reef in Queensland, Australia, the southeastern waters of Papua Bay in Papua New Guinea, and the northwestern waters of Papua Province in Indonesia. Overall, only about 18.67% of the mollusk priority protection areas were covered by existing MPAs. On the other hand, the regions most threatened by fishing pressure were mainly distributed in the South China Sea, Beibu Gulf, southwestern Papua Province in Indonesia, and the northeastern coastal waters of Australia (Figure 5f). Further comparative analysis of the spatial distribution of existing MPAs and fishing pressure hotspots within mollusk priority protection areas revealed some overlap, meaning that some mollusk priority protection areas covered by existing MPAs were still under high fishing pressure. Typical overlapping areas included the Great Barrier Reef (Great Barrier Reef MPA), the Gulf of Carpentaria (Gulf of Carpentaria MPA), Oceanic Shoals (Oceanic Shoals MPA), and the Kimberley Marine Protected Area (Kimberley MPA) along the Australian coast.

## 4 Discussion

### 4.1 The Relationship Between Biodiversity and Habitat Heterogeneity

Mollusk diversity in the Indo‐Pacific Convergence Zone (IPCZ) exhibited significant spatial heterogeneity, with distributions mainly concentrated in coastal areas, particularly where key biogenic habitats such as coral reefs, mangroves, and seagrass beds were widely present. Regions such as the Philippines, Indonesia, and Papua New Guinea showed high sampling effort and species richness of mollusks (Figure 3a,d). For instance, a single 1° × 1° grid cell in southern Luzon, Philippines, recorded as many as 741 species (Figure 3d and Table S2), which was likely closely related to the high coverage of key biogenic habitats in these areas (Barrientos‐Luján et al. 2021). The spatial distribution of areas with high Shannon‐Wiener index values in regions such as the Philippines and Papua New Guinea further supported the notion that habitat heterogeneity promoted community diversity (Figure 3g–i). By expanding ecological niche space and enhancing species differentiation, such heterogeneity helps maintain high levels of biodiversity (Tews et al. 2003). In addition, the IPCZ is also characterized by multi‐scale ocean dynamical processes, including precipitation, temperature, and nutrient availability variations driven by the Intertropical Convergence Zone (ITCZ) and related climatic phenomena. These environmental gradients create diverse habitats that support high species richness and endemism among mollusks and other marine organisms (Zhang et al. 2020; Du et al. 2023; Qian et al. 2024).

The IPCZ encompasses a range of marine environments, from coral reefs and seagrass beds to soft sediment bottoms, each providing distinct niches. This habitat heterogeneity enables different mollusk species with varying ecological needs to coexist, thereby increasing overall biodiversity (Wang et al. 2025). In this study, coral reefs covered approximately 23% of the Indo‐Pacific Convergence Zone (IPCZ), while mangroves and seagrass beds accounted for 19.7% and 3.8%, respectively. Among these key biogenic habitats, around 45.5% of the area featured a combination of two habitat types, and 10.4% supported the coexistence of all three (Table S2). Such multi‐habitat configurations significantly enhanced the complexity of local ecosystems. Regions such as the Philippines, Indonesia, and Australia provide diverse reproductive, foraging, and sheltering environments for mollusks with different ecological traits, thereby promoting species coexistence and reproduction (Badgley et al. 2017; Costello and Chaudhary 2017). Mollusk communities in these areas were not only species‐rich but also exhibited more balanced and stable community structures. Moreover, the IPCZ hosted several Critically Endangered (CR) and Endangered (EN) species listed by the IUCN, particularly among Gastropoda and Bivalvia, which are mainly distributed in regions with high habitat heterogeneity. This highlights that changes or degradation in biogenic habitat quality may directly threaten the survival of these vulnerable species (Ceccarelli et al. 2024; Polidoro et al. 2010; Unsworth and Cullen 2010).

In summary, the spatial patterns of mollusk diversity in the Indo‐Pacific Convergence Zone are largely influenced by biogenic habitat heterogeneity. The diversity and composite distribution of key ecosystems—such as coral reefs, mangroves, and seagrass beds—provide relatively stable habitat foundations for species occupying different ecological niches and play a crucial role in maintaining regional biodiversity. Future conservation efforts should prioritize core habitats with high heterogeneity, which are increasingly threatened by overfishing, coastal development, and climate change, in order to effectively preserve mollusk diversity and enhance ecosystem stability.

### 4.2 The Importance of Priority Conservation Areas for Mollusks

Mollusks are widely distributed and constitute an important component of marine ecosystems. They occupy a key position in the food chain and play an irreplaceable role in maintaining biodiversity and regulating ecological balance (Gutiérrez et al. 2003; Oehlmann and Schulte‐Oehlmann 2003). Compared to plankton, benthic mollusks tend to have more restricted geographic distributions, and they generally possess lower mobility than other large marine animals, such as marine mammals. As a result, priority conservation areas established based on their distribution ranges can remain effective over the long term without frequent adjustments due to migratory behavior. This provides greater stability and sustainability for mollusk conservation. The IPCZ itself contains a variety of mollusk species with diverse life histories, morphologies, and physiological traits. Protecting key areas is essential for maintaining this genetic and functional diversity, crucial for adapting to environmental changes, and ensuring long‐term species survival (Weng et al. 2025).

The priority conservation areas identified in this study were primarily concentrated in the coastal waters of the Philippines, Indonesia, Papua New Guinea, Australia, and the Solomon Islands (Figure 5d). Among them, several regions around Luzon Island, the Visayas, and Mindanao in the Philippines, as well as areas near the Solomon Islands, were recognized as the most important conservation sites. The priority areas identified in this study show a certain degree of similarity to the spatial patterns of biodiversity hotspots reported in previous research (Asaad et al. 2018; Sanciangco et al. 2013).

As early as 1988, renowned biodiversity and conservation biologist Norman Myers introduced the concept of “Biodiversity Hotspots.” He highlighted that although hotspot ecosystems occupy relatively small ecological space, they harbor remarkably high levels of species diversity (Myers 1988). In this study, priority conservation hotspots were identified based on sampling effort, species richness, and the Shannon‐Wiener index of mollusks and their biogenic habitats. Although these areas accounted for only approximately 2.33% of the total study region, they encompassed 10,615 mollusk occurrence records, 1268 threatened mollusk occurrence records, and 2082 biogenic habitat records. Moreover, these areas contained 1533 mollusk species (47.86%), 139 threatened mollusk species (58.90%), and 18.28% of the area supported the coexistence of all three key biogenic habitats (Table S2).

In conclusion, although the identified priority conservation areas occupied a relatively small spatial extent, they supported a rich array of species and provided critical ecosystem services. Protecting these biodiversity hotspot regions is not only essential for maintaining global ecological balance but also plays a pivotal role in mitigating biodiversity loss. Additionally, the results of this study revealed weak spatial correlations among the three composite indices, suggesting limited interactions between different metrics (Figure 6). This finding underscores the need to integrate various biodiversity indicators and ecological factors when assessing biodiversity hotspots to ensure a more thorough understanding and more effective conservation efforts.

### 4.3 Climate Adaptation Needs to Be Incorporated Into Conservation Planning

Marine mollusk biodiversity depends on a complex interplay of environmental factors, including water quality, temperature, salinity, pH, habitat type, and food availability. These factors influence species distribution, abundance, and community composition. In this study, we systematically evaluated the influence of environmental factors on marine mollusk biodiversity. The results indicated that primary productivity and bathymetry are the most critical determinants (Figure 7a,c,e). Areas with high productivity typically provide abundant food resources, which are conducive to sustaining diverse mollusk communities (Cecchetto et al. 2024). This finding aligns with previous studies, highlighting the vital role of primary productivity in supporting energy flow and regulating species distributions within marine ecosystems (Cecchetto et al. 2024; Costello and Chaudhary 2017). Bathymetry, on the other hand, directly influences mollusk habitats and population dynamics by affecting light availability, temperature stratification, and oxygen concentration (Ayers et al. 2014). Although temperature contributed relatively less to biodiversity indices in this study, its consistently negative effect suggested that rising temperatures could pose a potential threat to mollusk communities (Figure 7b,d,f). Elevated seawater temperatures may exceed the thermal tolerance thresholds of many mollusk species, leading to physiological stress, reduced feeding and growth, and increased mortality (Matoo et al. 2021; Seuront et al. 2019). This result implies that, under ongoing global warming, the spatial distribution patterns of mollusks in the Indo‐Pacific Convergence Zone may undergo substantial changes, potentially leading to adverse impacts on regional biodiversity.

Currently, only 18.7% of critical biodiversity areas are effectively protected globally, highlighting the urgent need to incorporate climate adaptability indicators into protected area management standards through policy tools such as the “National Climate Change Adaptation Strategy 2035 of China.” As global climate change intensifies, understanding and predicting the driving mechanisms of environmental factors is crucial for developing scientifically sound and effective conservation strategies. The findings of this study provide a theoretical foundation for the conservation and management of marine mollusks. Moving forward, it is essential to focus not only on strengthening the synergistic mechanisms between climate change and international marine conventions, but also on incorporating local ethics, anthropogenic activities, monitoring, and policy frameworks into an integrated marine protection system to achieve more effective biodiversity conservation.

### 4.4 Optimization of the Mollusk Conservation Network

Improving a mollusk conservation network in the IPCZ requires integrating ecological, spatial, and socio‐economic factors to ensure adequate biodiversity protection, functional connectivity, and sustainable utilization. This study found that only approximately 18.67% of the marine mollusk priority conservation areas were covered by existing Marine Protected Areas (MPAs), indicating that over 80% of the critical areas remained inadequately protected, reflecting a significant gap in the conservation of marine mollusks in the Indo‐Pacific Convergence Zone (Figure 5e). This protection gap manifested in two main aspects. First, the total coverage area of existing protected areas was relatively small, and their spatial distribution was highly uneven. For example, although many MPAs were distributed around the Philippines, most were small and fragmented, making it difficult to establish systematic protection of critical areas. To improve conservation effectiveness, it is recommended to expand the coverage of MPAs in these biodiversity hotspot areas and enhance their spatial connectivity to strengthen ecosystem integrity and resilience. Secondly, in some priority conservation areas, protection and fishing ban measures are difficult to enforce effectively. For example, the Coral Sea area near northeastern Australia and Papua New Guinea, despite having established MPAs, remains a high‐intensity fishing area, facing a significant conflict between resource development and ecological protection, which weakens the effectiveness of the protected areas (Figure 5f). To address this challenge, it is recommended to designate targeted protection zones around these areas and implement stricter and more scientifically sound fisheries management measures. Even if a complete fishing ban cannot be achieved, it is essential to reduce the impact of human activities on mollusk populations through measures such as limiting fishing intensity and optimizing management strategies, ensuring the sustainable development of ecosystems in these regions.

The Indo‐Pacific Convergence Zone, a global hotspot for marine biodiversity, is facing significant fishing pressure, particularly on marine mollusks. Although fishing bans provide temporary relief, their impact is often limited in both time and space, failing to substantially reduce overall fishing intensity in the region. This issue aligns with existing studies on the conflict between fisheries and ecological protection (Zhang et al. 2024), highlighting the need for more targeted and refined fisheries management strategies that address both spatial and temporal dimensions. To protect endangered species in this area, ecosystem‐based fisheries management is essential. Effective management can be achieved through dynamic spatial governance measures, such as establishing transnational ecological corridors and seasonal fishing bans, alongside technological innovations like the Ecopath model to optimize quotas and develop selective fishing gear. Furthermore, multinational institutional cooperation is crucial for aligning with the BBNJ agreement and regional fisheries organizations. These approaches will not only safeguard key habitats, such as the Coral Triangle, but also ensure the sustainable development of fisheries in the region. Implementing all those strategies can also enhance the mollusk conservation network in the IPCZ, improving biodiversity protection, ecosystem resilience, and sustainable use, effectively addressing both ecological and human factors.

### 4.5 Research Limitations and Future Directions

Although this study provides a preliminary analysis for the conservation of marine mollusk diversity in the Indo‐Pacific Convergence Zone, there are still certain limitations. The acquisition of biological samples is fundamental to biodiversity research and conservation efforts. However, due to restrictions or even bans on foreign research vessels entering some key countries in the Indo‐Pacific Convergence Zone, the collection of biological samples in certain areas has been challenging. This has, to some extent, resulted in limitations in mollusk distribution data, which may affect the spatial representativeness of the study's results, particularly in under‐surveyed areas. Therefore, future research could enhance the integrity and spatial coverage of data by strengthening international cooperation, expanding data collection efforts, and integrating remote sensing and ecological modeling techniques, thereby providing more scientific support for the conservation of marine mollusks in the region.

While our study primarily focused on taxonomic and community‐based diversity metrics, incorporating functional diversity could substantially enhance our understanding of molluscan biodiversity patterns. At present, the lack of standardized and comprehensive trait data across the IPCZ constrains such analyses. Therefore, we highlight the development of functional trait databases as a priority for future research. Similarly, available habitat data are largely provided as polygon‐based layers (e.g., coral reefs, seagrass beds, and soft sediments), which represent habitat extent but do not capture continuous spatial heterogeneity or structural complexity. As a result, habitat heterogeneity was not incorporated as a predictor in our SHAP analysis. Subsequent studies using high‐resolution, raster‐based environmental metrics could help better quantify habitat heterogeneity and improve mechanistic understanding of its influence on mollusk biodiversity.

It is noteworthy that, within the Indo‐Pacific Convergence Zone, a total of 173 mollusk species were classified as “Least Concern” according to the IUCN Red List of Threatened Species (Table S1). This phenomenon reflects that, despite the crucial role mollusks play in maintaining marine ecosystem functions, their endangered status has not received the attention it deserves. Future efforts should focus on strengthening the conservation research and investment in this group, promoting systematic endangered species assessments, and developing and implementing conservation management measures to ensure the sustainable survival and ecological function stability of mollusk populations in marine ecosystems.

## 5 Conclusion

This study integrates multisource data, including species distributions, habitats, environmental drivers, MPAs, and fishing pressure, to systematically identify priority conservation areas for marine mollusks in the IPCZ, going beyond traditional species‐richness‐based approaches. Key innovations include using SHAP values to quantify environmental impacts and identifying multi‐habitat zones (e.g., coral‐mangrove combinations) as vital, yet often overlooked, biodiversity hubs. The results highlight 11 priority areas, containing 47.9% of mollusk species and 58.9% of threatened taxa, which remain inadequately protected (with only 18.7% MPA coverage) and face intense fishing pressure (72.6% of total effort). Primary productivity and bathymetry were found to be the dominant environmental drivers, while rising temperatures posed significant threats. The findings highlight the shortcomings of current conservation measures and suggest expanding the protection of high‐diversity habitats, implementing ecosystem‐based fisheries management, and incorporating climate adaptability into conservation planning. These measures will effectively enhance the protection and resilience of marine ecosystems in the IPCZ region.

## Author Contributions

**Xianshui Lai:** formal analysis (lead), methodology (lead), resources (lead), software (lead), visualization (lead), writing – review and editing (lead). **Tingting Li:** methodology (equal), software (equal), visualization (equal). **Bailin Cong:** data curation (equal), formal analysis (equal), visualization (equal). **Pipit Pitriana:** conceptualization (equal), writing – review and editing (equal). **Bei Wang:** data curation (equal), formal analysis (equal), validation (equal). **Linlin Zhao:** data curation (equal), formal analysis (equal), funding acquisition (equal), validation (lead). **Shenghao Liu:** conceptualization (lead), funding acquisition (lead), project administration (lead), supervision (lead), writing – review and editing (lead).

## Conflicts of Interest

The authors declare no conflicts of interest.

## Supporting information


**Figure S1:** Taxonomic hierarchy and distribution density of mollusks in the study area. The central node represents the phylum mollusks, with branches in different colors corresponding to different classes. The terminal nodes of each branch represent specific orders. The size of each node reflects the number of distribution points, with larger circles indicating higher distribution density.
**Figure S2:** Distribution patterns of mollusks and marine ecosystems in Indo‐Pacific convergent zone according to sampling effort (SE), species richness (SR), and Shannon‐Wiener index (SN). (a) SE of all molluscan species (ALL); (b) SE of threatened molluscan species (TSP); (c) SE of coral, seagrass and mangrove (CSM); (d) SR of all molluscan species (ALL); (e) SR of threatened molluscan species (TSP); (f) SR of coral, seagrass and mangrove (CSM); (g) SN of all molluscan species (ALL); (h) SN of threatened molluscan species (TSP); (i) SN of coral, seagrass and mangrove (CSM).
**Figure S3:** Marine fisheries catch effort in the Indo‐Pacific convergence zone (2021–2023). Mollusk fishing pressure refers to the catch effort targeting mollusks; non‐mollusk fishing pressure refers to the catch effort targeting other marine organisms; ambiguous fishing pressure refers to catch effort with unclear target species.

**Table S1:** List of mollusks of conservation concern in the IPCZ based on IUCN Red List and the CITES.

**Table S2:** Distribution of biological and hotspots in 1‐degree cells.

## Data Availability

All the required data is uploaded as Supporting Information.
