# Supplementary material for: Biodiversity and Conservation of Marine Mollusks in the Indo‐Pacific Convergence Zone
Source: Ecol Evol. 2025 Nov 12;15(11):e72364. doi: 10.1002/ece3.72364 (PMC12611349; doi:10.1002/ece3.72364)
Supplement: Supplementary file 1 — Figure S1: Taxonomic hierarchy and distribution density of mollusks in the study area. The central node represents the phylum mollusks, with branches in different colors corresponding to different classes. The terminal nodes of each branch represent specific orders. The size of each node reflects the number of distribution points, with larger circles indicating higher distribution density. Figure S2: Distribution patterns of mollusks and marine ecosystems in Indo‐Pacific convergent zone according to sampling effort (SE), species richness (SR), and Shannon‐Wiener index (SN). (a) SE of all molluscan species (ALL); (b) SE of threatened molluscan species (TSP); (c) SE of coral, seagrass and mangrove (CSM); (d) SR of all molluscan species (ALL); (e) SR of threatened molluscan species (TSP); (f) SR of coral, seagrass and mangrove (CSM); (g) SN of all molluscan species (ALL); (h) SN of threatened molluscan species (TSP); (i) SN of coral, seagrass and mangrove (CSM). Figure S3: Marine fisheries catch effort in the Indo‐Pacific convergence zone (2021–2023). Mollusk fishing pressure refers to the catch effort targeting mollusks; non‐mollusk fishing pressure refers to the catch effort targeting other marine organisms; ambiguous fishing pressure refers to catch effort with unclear target species. [file ECE3-15-e72364-s001.docx]

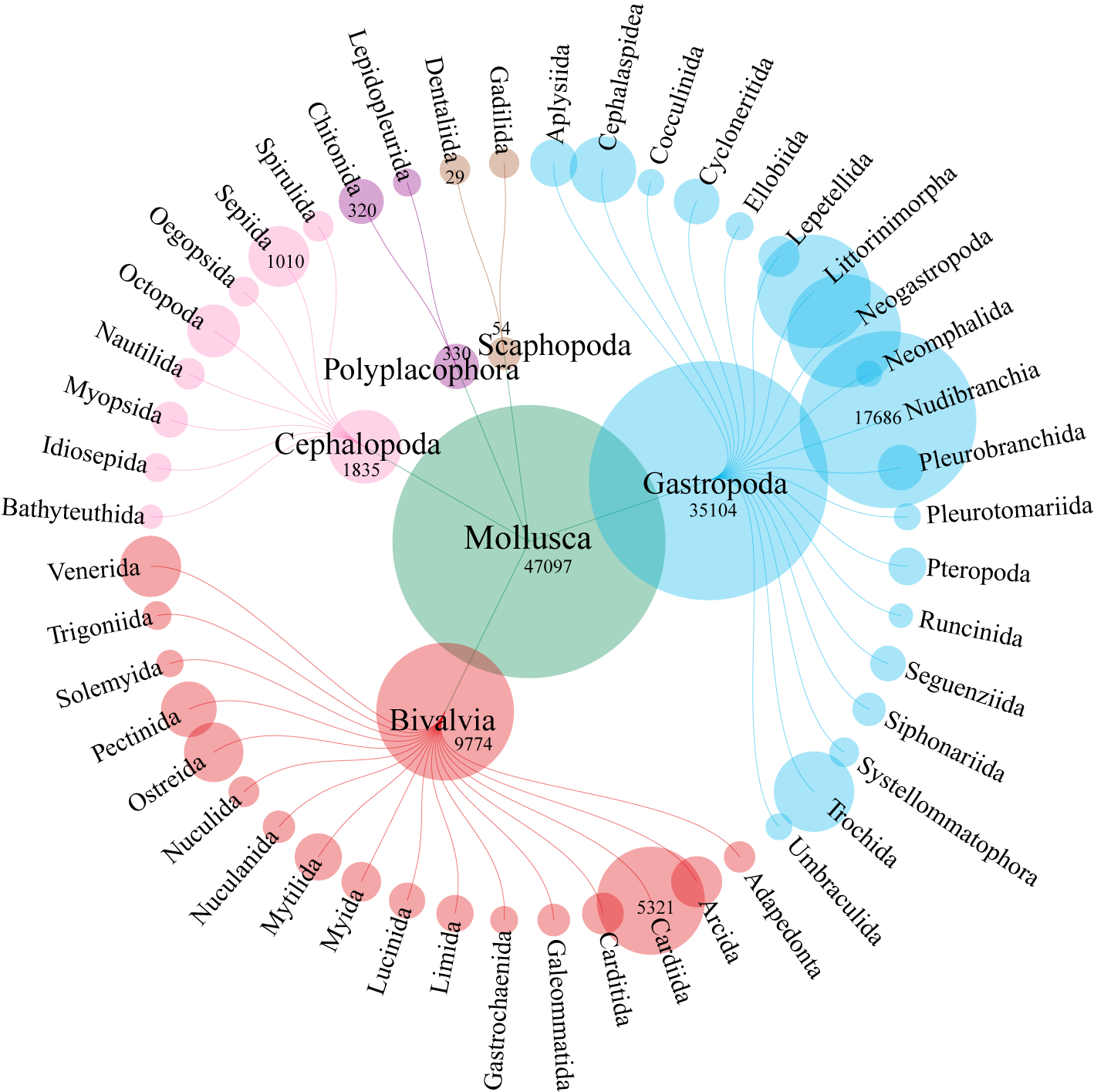


Fig. S1. Taxonomic Hierarchy and Distribution Density of Mollusca in the Study Area. The central node represents the phylum Mollusca, with branches in different colors corresponding to different classes. The terminal nodes of each branch represent specific orders. The size of each node reflects the number of distribution points, with larger circles indicating higher distribution density.


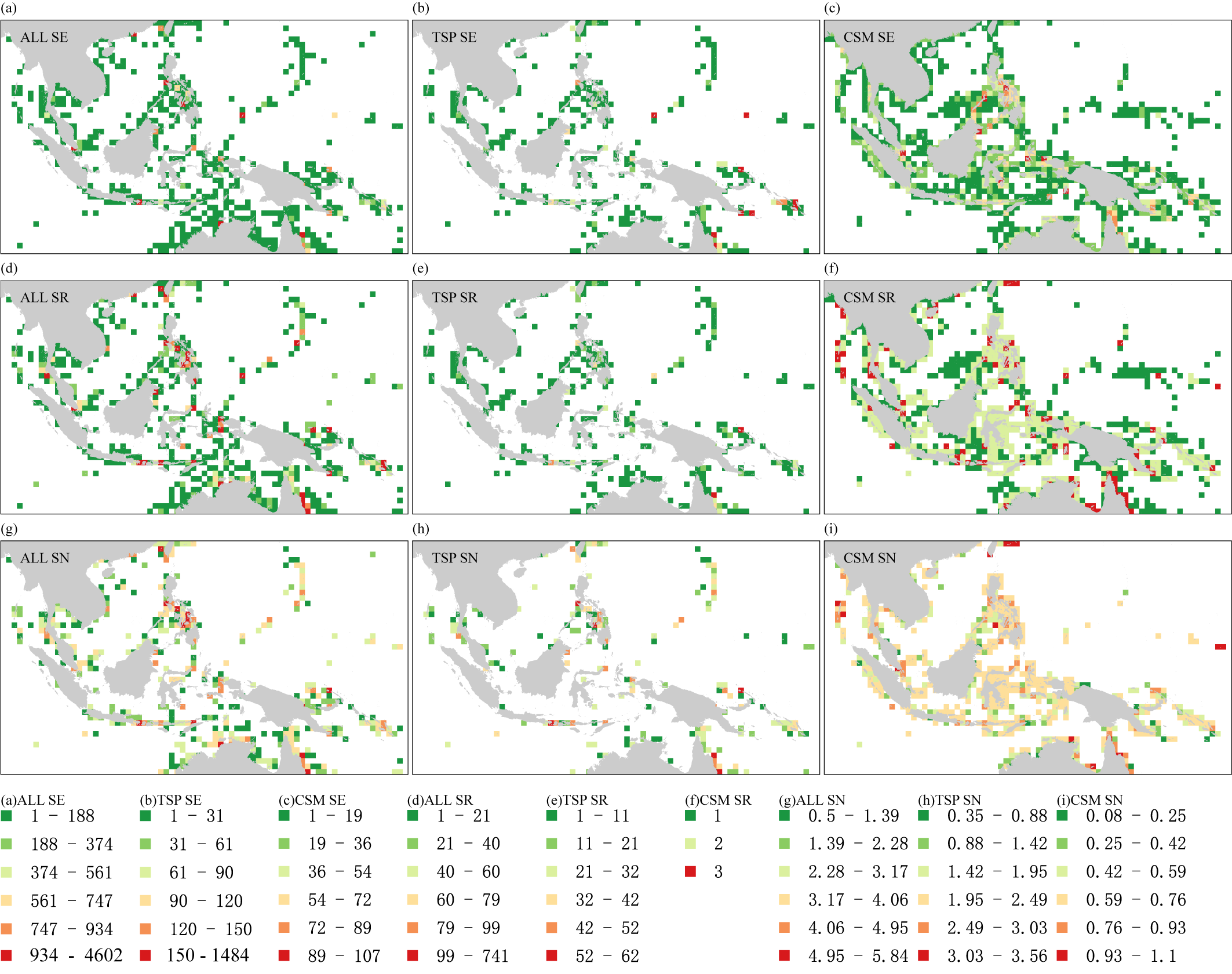


Fig. S2. Distribution patterns of Mollusca and marine ecosystems in Indo-Pacific Convergent Zone according to sampling effort (SE), species richness (SR), and Shannon Wiener index (SN). (a) SE of all molluscan species (ALL); (b) SE of threatened molluscan species (TSP); (c) SE of coral, seagrass and mangrove (CSM); (d) SR of all molluscan species (ALL); (e) SR of threatened molluscan species (TSP); (f) SR of coral, seagrass and mangrove (CSM); (g) SN of all molluscan species (ALL); (h) SN of threatened molluscan species (TSP); (i) SN of coral, seagrass and mangrove (CSM).


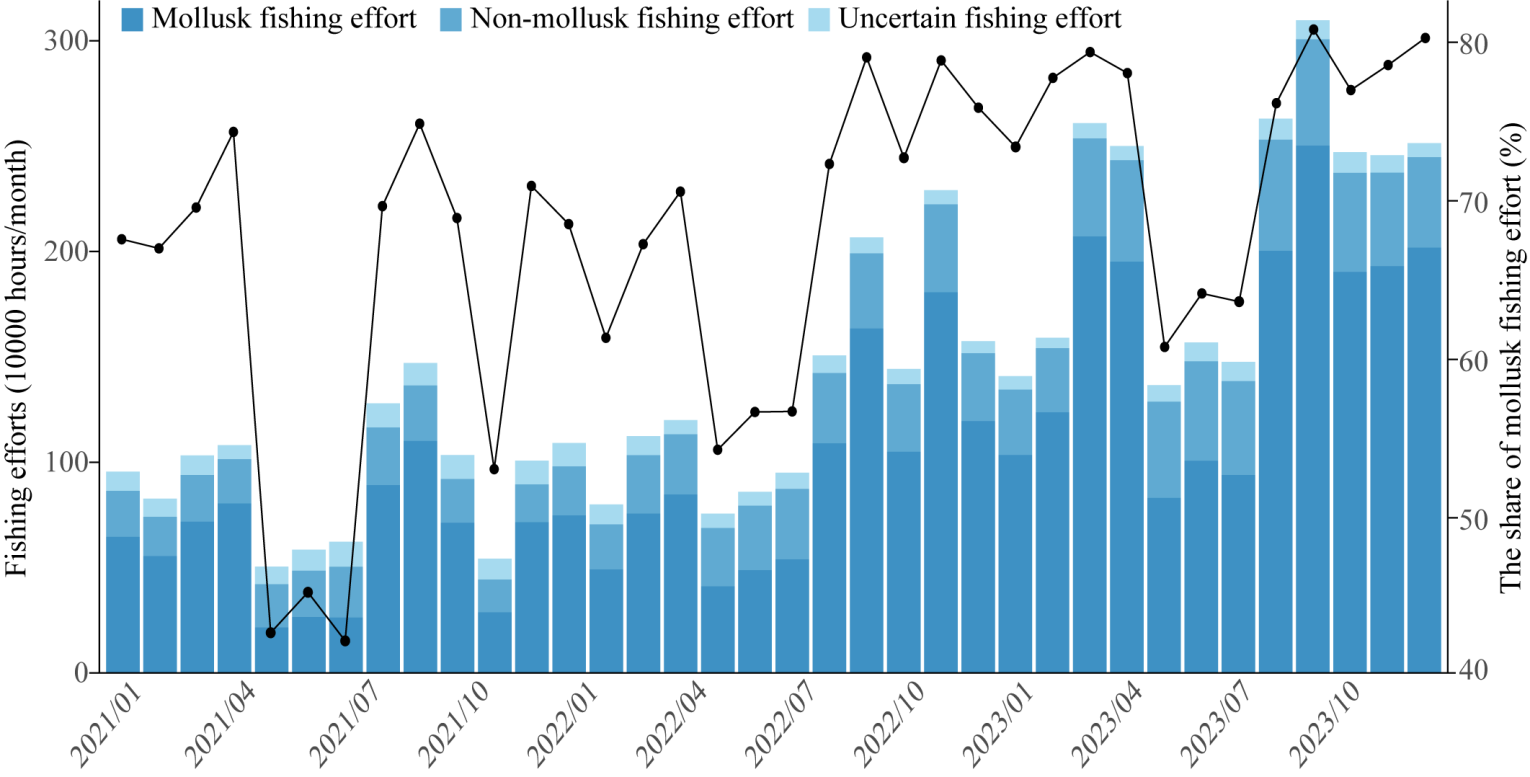


Fig. S3. Marine Fisheries Catch Effort in the Indo-Pacific Convergence Zone (2021-2023). Mollusk fishing pressure refers to the catch effort targeting mollusks; non-mollusk fishing pressure refers to the catch effort targeting other marine organisms; ambiguous fishing pressure refers to catch effort with unclear target species.
